# Cognitive correlates of repetitive transcranial magnetic stimulation (rTMS) in treatment-resistant depression- a pilot study

**DOI:** 10.1186/1471-244X-12-163

**Published:** 2012-10-02

**Authors:** Karina K Kedzior, Vikram Rajput, Greg Price, Joseph Lee, Mathew Martin-Iverson

**Affiliations:** 1 School of Humanities and Social Sciences, Jacobs University Bremen, Campus Ring 1, Bremen, 28759, Germany; 2 Clinical Neurophysiology Unit, Graylands Hospital, John XXIII Avenue, Mt Claremont, Perth, 6010, Australia; 3 Pharmacology & Anaesthesiology Unit, School of Medicine & Pharmacology, Faculty of Medicine, Dentistry & Health Sciences, The University of Western Australia, 35 Stirling Highway, Crawley, Perth, 6009, Australia

**Keywords:** Repetitive transcranial magnetic stimulation (rTMS), Treatment-resistant depression, Modified concept-shifting task (mCST), Left dorsolateral prefrontal cortex (DLPFC)

## Abstract

**Background:**

The aim of the current study was to investigate the cognitive correlates of repetitive transcranial magnetic stimulation (rTMS) in 10 treatment-resistant depression patients.

**Methods:**

Patients received forty 20-min sessions of fast-frequency (10 Hz) rTMS of the left dorsolateral prefrontal cortex (DLPFC) over 20 days. Concept-shift ability (accuracy and duration of performance) was assessed daily with a Modified Concept-Shifting Task (mCST) in patients and in eight healthy volunteers. General cognitive functioning test (Repeatable Battery for the Assessment of Neuropsychological Status; RBANS), Beck Depression Inventory (BDI) and Hamilton Depression Rating Scale (HAM-D) were applied before the first and after the last rTMS.

**Results:**

Compared to before rTMS on the first 10 days, the patients performed the mCST significantly more accurately after rTMS on the last 10 days (*p* < .001, partial eta squared=.78) while the same comparison in healthy volunteers was not statistically significant (*p* = .256, partial eta squared=.18). A significant improvement in immediate memory on RBANS and reduction in BDI and HAM-D scores were also observed after the last compared to before the first rTMS.

**Conclusion:**

The rTMS is associated with an improvement in selective cognitive functions that is not explained by practice effects on tasks administered repeatedly.

**Trial registration:**

Name: "Repetitive Transcranial Magnetic Stimulation (rTMS) in the treatment of depression, assessed with HAM-D over a four week period."

URL: www.actr.org.au

Registration number: ACTRN012605000145606

## Background

A repetitive transcranial magnetic stimulation (rTMS) is either a slow-frequency (≤1 Hz) or a fast-frequency (>1 Hz) non-invasive brain stimulation method [1]. A number of meta-analyses have shown that the rTMS appears to have antidepressive properties although the effect sizes were only modest, most likely due to different rTMS protocols adopted and different methods of meta-analysis used for (review see [2,3]). When focusing on the location and frequency of stimulation, it appears that such antidepressive properties are associated specifically with the left frontal fast-frequency rTMS [4]. The rTMS appears especially promising for approximately 15-20% of patients with major depression who fail to respond to standard pharmacotherapy and/or ECT [2] and thus are considered ‘treatment-resistant’ [5].

The antidepressive properties of the rTMS are often assessed by comparing severity of depression scores on standard instruments, such as the Hamilton Depression Rating Scale- 21 items, HAM-D [6] and the Beck Depression Inventory, BDI [7], before and after a period of the rTMS treatment that typically ranges between 5–20 sessions [8]. Since clinical depression is associated with decreased activation and blood flow in both the left and bilateral dorsolateral prefrontal cortex, DLPFC [9], another way of assessing the effectiveness of the rTMS is to compare various cognitive functions directly or indirectly controlled by the prefrontal cortex before and after the rTMS.

One task that can be used to investigate higher cognitive processes controlled by the DLPFC, including the ability to shift attention, the strategy to perform the test, visual recognition and scanning, and short and long-term memory, is the Modified Concept Shifting Task, mCST [10]. The mCST is a simple pen and paper example of a trail-making test that is cheap and easy to administer repeatedly. The major advantage of this task, compared to other standard cognitive tests, is that the accuracy of performance on the mCST was robust against practice effects when administered twice a day, separated by a 30 min break, for eight days in healthy humans [10]. While accuracy is not affected by practice, the duration of performance on the mCST might be affected by practice because, in the absence of any treatment, it was significantly shorter on the last day (day 8) compared to baseline (day 1) and after vs. before the break on each testing day [10]. Therefore, at least in terms of accuracy of performance, the mCST appears suitable for repetitive administration and any improvements in the accuracy could be attributed to treatment, such as the rTMS, rather than practice.

The aim of the current study was to investigate the cognitive changes associated with a fast-frequency rTMS of the left DLPFC in a pilot sample of patients with treatment-resistant major depression. It was hypothesised that if such a fast-frequency rTMS would directly or indirectly alter the DLPFC functioning then the patients would show an improvement in the accuracy and duration of performance on the mCST (after vs. before rTMS on each session and after the last vs. before the first rTMS). In addition, as a control measure, it was hypothesised that rTMS would reduce depression symptom severity on the HAM-D and the BDI and improve other cognitive functions, such as immediate memory, visuospatial/constructional ability, language, attention, and delayed memory assessed by a standard cognitive test, the Repeatable Battery for the Assessment of Neuropsychological Status, RBANS [11], as compared to baseline (before rTMS).

Due to the potential practice effects on the duration of performance on the mCST seen over eight days [10], a non-clinical healthy volunteer group was included in the current study to monitor their performance on the mCST concurrently to the patients for 20 days. The performance on the mCST over the first 8/20 days of testing has already been published as part of a larger data set for this *N* = 8 group [10] while the current study reports their performance on the task over the full length of experiment (20 days). It was hypothesised that, in the absence of any treatment, an improvement in the accuracy and/or duration of performance on the mCST in healthy volunteers would indicate presence of practice effects on the task either within-session (after vs. before a session break on each day) or on the last day (day 20) vs. baseline (day 1) of the experiment.

## Methods

### Participants

The study was approved by the Research Ethics Committees at the University of Western Australia and the North Metropolitan Area Mental Health Services in Perth, Australia. The study adopted a pilot, one treatment-group only design, similarly to other studies of cognitive performance after antipsychotic treatment in schizophrenia that also did not include a control sham group for (review see [12]). The participants were recruited from private and public mental health clinics of the North Metropolitan Area Health Service in Perth. Written informed consent was given by 11 patients with a DSM-IV and ICD-10 Australian Modification (ICD-10-AM) diagnosis of major depression, made by a research psychiatrist (JL) who was blind to the specific treatment protocol to be assigned to the patients. Non-response to treatment was defined as an unsatisfactory response to either standard pharmacotherapy (Tricyclics, MAOIs, SSRIs) and/or at least one course of the ECT based on referring clinician’s report and patients’ self-report. Standard pharmacotherapy was continued for ethical reasons and was stable for two weeks prior to and throughout the period of the experiment. According to a screening interview the participants had no history of epilepsy or seizures, current psychosis and/or substance dependence, stroke or major head trauma, or implants which could affect the rTMS. One female patient stopped the rTMS after 14 days of treatment due to a deteriorating psychological condition. Therefore, the final analyses were conducted using data of 10 patients (5 male; *M* ± *SD* age of 43 ± 9 years, range 24–54 years). The patients were monitored by the psychiatrist (JL) at baseline (day 1 of rTMS), during week 2 sessions, and on the last day of testing (day 20).

To further investigate the effects of practice on the mCST, the task was also administered to eight task-naïve healthy volunteers (6 male; *M* ± *SD* age of 39 ± 15 years, range 18–53) recruited from staff not involved in the project and relatives of the project’s personnel. Since the healthy volunteers were not matched with the patients they are referred to as ‘healthy volunteers’ rather than ‘controls’. The healthy volunteers were considered as participants in a parallel sub-study and thus all their data were analysed separately from that of the patients.

### The rTMS procedure

The rTMS consisted of a fast-frequency (10 Hz) stimulation of the left DLPFC. The participants were seated comfortably at a 45-degree angle during the rTMS procedure. The F3 position in the ten-twenty system was used to locate the left DLPFC [13]. All stimulation was performed with intensity equal to 100% motor threshold [14]. The patients underwent 20 rTMS sessions of 20 min each administered at the same time over a period of consecutive 20 working days. The stimulation in randomly selected five patients consisted of 40 pulse trains of 5 s at 10 Hz, and separated by 25 s inter-train intervals, totaling 2000 pulses per session. The other five patients received three stimuli every time a computer-based EEG-pattern recognition device detected a combination of alpha (8–13 Hz) and/or beta (13–20 Hz) oscillations at the Pz location with a 15 s inter-train interval averaging approximately 50 stimuli/30 s, totalling approximately 2000 pulses. This so-called interactive technique had shown a trend towards improving the clinical efficacy compared to the standard rTMS in treatment-resistant patients with depression [15].

### Cognitive assessment- mCST on each day (Day 1–20)

The details of the mCST task have been described elsewhere [10]. Briefly, the mCST was administered on paper and consisted of eight trials. There was no practice trial. On each trial participants were verbally instructed to cross out, as fast as possible, 10 numbers or 10 letters randomly selected from a set of 26 numbers (1–26) or 26 letters (A-Z) respectively, either in ascending (numerical or alphabetical) or descending order. All 10 stimuli (letters or numbers) were presented simultaneously in each trial.

The task was pseudo-randomised in that the set of numbers or letters alternated among all trials (for example, if trial 1 consisted of letters then trial 2 consisted of numbers followed by letters and so on), while the order of number and letter sets always remained the same for every participant. The task instruction remained constant on trials 1–4 (for example, ‘cross out the letters or numbers in ascending- alphabetical or numerical- order’) and was then reversed before trial 5 (for example, ‘now cross out the letters or numbers in *descending-* reverse-alphabetical or from highest to lowest- order’) and remained constant on trials 5–8. Therefore, the mCST measures a concept shift that includes alternating stimuli (either letters or numbers) from trial 1 to trial 8 and, in addition, a shift in the strategy needed to complete the task (following the instruction regarding the ascending or the descending order) on trial 5 onwards.

The performance on the mCST was quantified in terms of the accuracy of performance (% accurate responses) and the total duration of performance (between crossing out the first and the last stimuli, in s) on trials 1, 5 and 8. The participants were not required to correct their errors. The error in accuracy was recorded if a participant started with an incorrect item, skipped an item, or ignored the instruction. For example, if G was crossed out before C on a letter trial in ascending order and all other letters were crossed out correctly then the accuracy on this trial was 9/10 or 90% [10].

All participants completed the mCST daily before and after the rTMS (patients) or before and after a 30-min session break (healthy volunteers) that was equivalent to the time taken to administer the rTMS in patients, on 20 concurrent working days. The healthy volunteers were free to do what they wished during the break.

The duration and accuracy of performance were recorded on trials 1, 5 and 8 in all participants. Consequently the mean accuracy and duration of performance used in the statistical analyses were computed from these three trials, separately for both variables (duration and accuracy), and separately for trials before and after treatment (rTMS in patients) or before and after break (in healthy volunteers).

### Cognitive and clinical assessment- baseline (Day 1) vs. Day 20

#### General cognitive functioning (RBANS)

The RBANS [11] task (administered by VR) was used to assess the immediate memory, visuospatial/constructional ability, language, attention, and delayed memory at baseline (day 1, before rTMS) and at the end of experiment (day 20, after rTMS) in patients only. The total score for each function was translated into a percentile score using an index table which contains age-matched data from a large cohort of participants (the higher the score the better the cognitive function). An alternate form of RBANS was used for the second assessment to reduce practice effects.

#### Depression severity (HAM-D and BDI)

The HAM-D [6] and BDI [7] were administered by a psychiatrist (JL) to determine the severity of depression scores at baseline (day 1, before rTMS) and at the end of experiment (day 20, after rTMS) in patients.

## Results

The mean duration and the mean accuracy of performance on the mCST were analysed separately in SPSS 20.0 using the following comparisons:

1. trial 1 day 1 (before the first rTMS) vs. trial 8 day 20 (after the last rTMS).

2. before vs. after rTMS regardless of block.

3. the first 10 days (block 1) vs. the last 10 days (block 2) of testing regardless of treatment.

4. the first 10 days before rTMS vs. the last 10 days after rTMS.

Comparison 1 was tested using the non-parametric Wilcoxon Signed-Rank *T*-test (equivalent to the paired-samples *t*-test) due to the violation of normality of scores in some of the variables included in these analyses. In case any participants missed experimental days 1 or 20 the performance on their individual first and last day was taken into account when computing the T-tests (2/10 patients missed day 1 and one other patient missed day 20 of the experiment).

Comparisons 2 and 3 were tested using a repeated measures ANOVA with two within-subject factors (TREATMENT with two levels: before vs. after rTMS; BLOCK with two levels: 1 = first 10 days of rTMS vs. 2 = last 10 days of rTMS). Comparison 4 was tested using a repeated measures ANOVA with one within-subject factor (TIME with two levels: before rTMS on the first 10 days vs. after rTMS on the last 10 days). The main effects and TREATMENT × BLOCK interactions were further investigated using pairwise comparisons with Sidak’s correction for multiple comparisons. In general, ANOVA is robust against any violations of univariate normality of scores. Furthermore, the Sidak’s posthoc test was chosen because it provides an optimised balance between Type I and Type II errors compared to the two other tests available under the pairwise comparisons options in SPSS- the Least Significant Difference (LSD) Test (equivalent to an uncorrected t-test) with a high Type I error chances and Bonferroni’s test with low power and high Type II error chances.

The same analyses as above were also conducted separately for the healthy volunteers using a factor BREAK (with two levels: before vs. after the session break) instead of a factor TREATMENT (rTMS) used in patients.

### Duration of performance on the mCST

The pattern of results regarding the duration of performance on the mCST differed between patients and healthy volunteers according to comparisons 1 and 2. Specifically, the patients performed the mCST significantly faster (*p* = .047) on the last vs. the first trial of the experiment (Table 1 Duration). The same comparison was not statistically significant (*p* = .161) in the healthy volunteers.

**Table 1:** **Median duration and accuracy of performance on the mCST before the first (trial 1, day 1) vs. after the last (trial 8, day 20) rTMS in patients (*N*** **= 10) or a break (equivalent to the time taken to administer rTMS in patients) in healthy volunteers (*N*** **= 8)**

**Score (Median; range)**	**Before first rTMS or break**	**After last rTMS or break**	*** T (z)***	***p***_***two-tailed***_	*** r***
**DURATION**					
Patient	24 (14–48)	20 (10–29)	8.0 (−2.0)	.047*	**-.45**
Healthy volunteer	16 (11–19)	15 (8–19)	8.0 (−1.4)	.161	-.35
**ACCURACY**					
Patient	90 (80–100)	100 (90–100)	15.0 (2.1)	.038*	**.47**
Healthy volunteer	80 (70–100)	100 (80–100)	10.0 (1.9)	.059	.48

mCST- Modified Concept-Shifting Task; *T*- Wilcoxon Signed-Rank *T* Test; The effect size *r* was calculated as *r* = *z*/√*N*, where *N* = a total number of measurements/case; *N* = 20 (patients) or *N* = 16 (healthy volunteers).

*p < .05.

Furthermore, the patients performed the mCST significantly faster (*p* = .040) after (*M ± SEM* of 17.6 ± .7 s) vs. before (18.0 ± .7 s) rTMS regardless of block (Table 2 Duration, main effect of TREATMENT). The same comparison was not statistically significant (*p* = .126) in the healthy volunteers (Table 2 Duration, main effect of BREAK).

**Table 2:** **The results of repeated measures ANOVAs comparing the mean duration or accuracy of performance on the mCST in patients (*N*** **= 10) and healthy volunteers (*N*** **= 8)**

**Effects**	***df; df***_***error***_	***F***	***p***_***two-tailed***_	***η***^***2***^_***part***_	**Power**
**DURATION**					
**PATIENT**					
TREATMENT	1; 9	5.7	.040*	**.39**	.57
BLOCK	1; 9	14.3	.004*	**.61**	.92
TREATMENT × BLOCK	1; 9	1.3	.276	.13	.18
**HEALTHY VOLUNTEER**					
BREAK	1; 7	3.0	.126	.30	.32
BLOCK	1; 7	41.9	<.001*	**.86**	1.00
BREAK × BLOCK	1; 7	3.9	.089	.36	.40
**PATIENT**					
TIME	1; 9	25.7	.001*	**.74**	.99
**HEALTHY VOLUNTEER**					
TIME	1; 7	23.0	.002*	**.77**	.98
**ACCURACY**					
**PATIENT**					
TREATMENT	1; 9	.2	.632	.03	.07
BLOCK	1; 9	21.2	.001*	**.70**	.98
TREATMENT × BLOCK	1; 9	.7	.432	.07	.11
**HEALTHY VOLUNTEER**					
BREAK	1; 7	.8	.406	.10	.12
BLOCK	1; 7	9.7	.017*	**.58**	.76
BREAK × BLOCK	1; 7	.001	.970	<.01	.05
**PATIENT**					
TIME	1; 9	31.4	<.001*	**.78**	1.00
**HEALTHY VOLUNTEER**					
TIME	1; 7	1.5	.256	.18	.19

*df*- degrees of freedom, *η*^*2*^_*part*_- partial eta squared (effect size). All ANOVAs were conducted using the following within-subject factors:

· TREATMENT: before vs. after rTMS.

· BLOCK: 1 = first 10 days vs. 2 = last 10 days of experiment.

· BREAK: before vs. after session break between the mCST administrations (equivalent to the time taken to administer rTMS in patients) in healthy volunteers.

· TIME: before rTMS or break on the first 10 days vs. after rTMS or break on the last 10 days of experiment.

*p < .05.

According to comparison 3 there was a significant main effect of BLOCK on the duration of performance on the mCST in both groups (Table 2 Duration). The patients performed the mCST significantly faster (*p* = .004) on the last 10 days (BLOCK 2; 16.9 ± .8 s) vs. the first 10 days (BLOCK 1; 18.8 ± .8 s) of the experiment regardless of treatment. Similarly, the healthy volunteers also performed the mCST significantly faster (*p* < .001) on BLOCK 2 (12.0 ± 1.0 s) vs. BLOCK 1 (13.8 ± 1.1 s) regardless of break.

**Figure 1:**
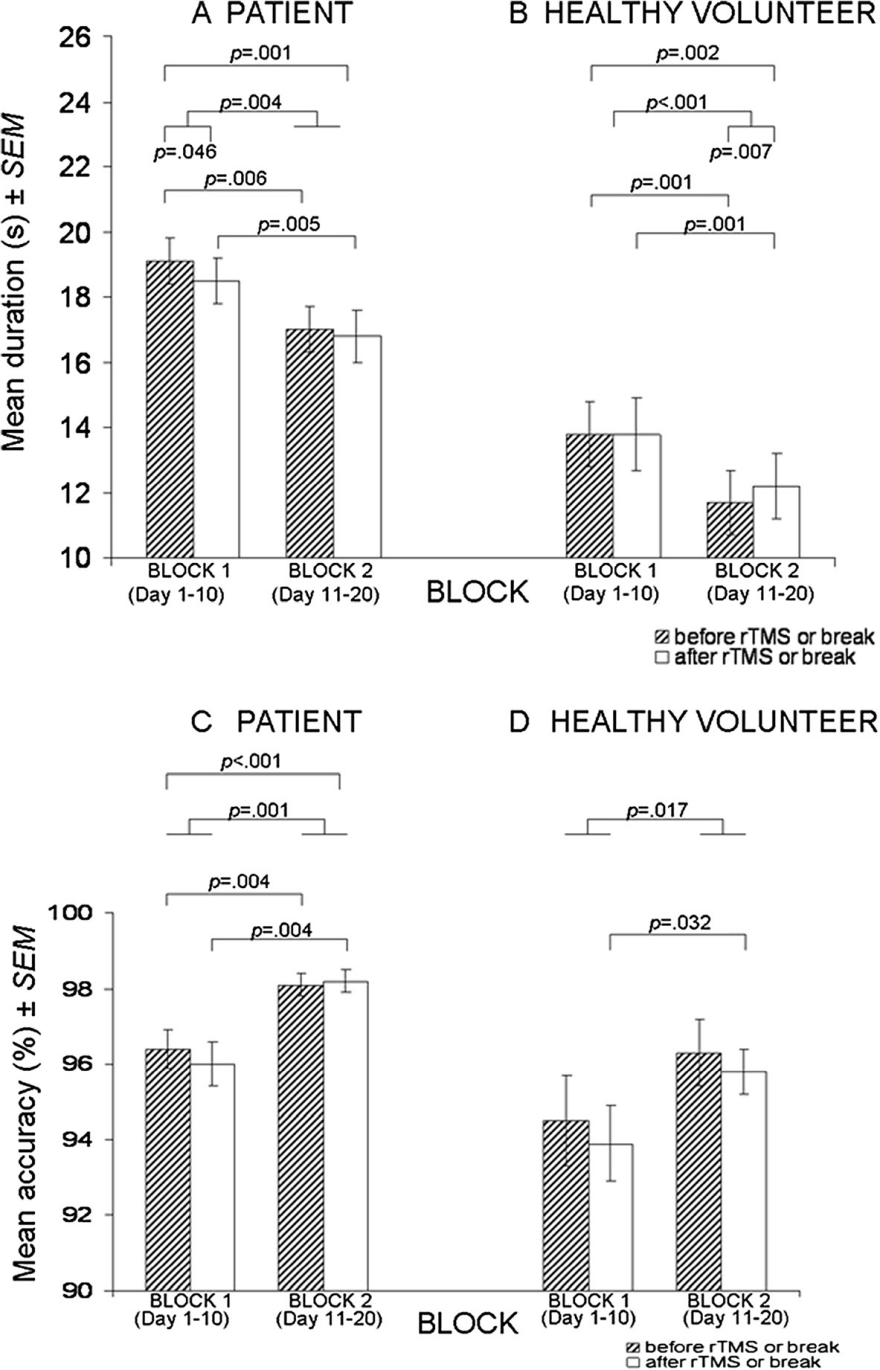
The mean duration and accuracy of performance on the mCST before and after rTMS in patients (A and C) or a break (equivalent to the time taken to administer rTMS in patients) in healthy volunteers (B and D) on block 1 (days 1–10) and block 2 (days 11–20) of the experiment.

When both TREATMENT (or BREAK) and BLOCK were taken into consideration, the pairwise comparisons showed that patients performed significantly faster after vs. before rTMS on BLOCK 1 only (Figure 1A; *p* = .046) while healthy volunteers performed significantly *slower* after vs. before the break on BLOCK 2 only (Figure 1B; *p* = .007). Furthermore, patients performed significantly faster on BLOCK 2 vs. BLOCK 1 before rTMS (Figure 1A; *p* = .006) and on BLOCK 2 vs. BLOCK 1 after rTMS (Figure 1A; *p* = .005). Like the patients, the healthy volunteers also performed significantly faster on BLOCK 2 vs. BLOCK 1 before the break and on BLOCK 2 vs. BLOCK 1 after the break (Figure 1B; both comparisons with *p* = .001).

Finally, according to comparison 4, all participants performed significantly faster on BLOCK 2 after rTMS (or break) vs. BLOCK 1 before rTMS (or break; Table 2 Duration, main effect of TIME; Figure 1A, *p* = .001 in patients; Figure 1B, *p* = .002 in healthy volunteers).

Regardless of statistical significance, the same comparisons involving duration of performance on the mCST had similar effect sizes (partial eta squared, *η*^*2*^_*part*_) in both groups (Tables 1 and 2 Duration). Thus, it is likely that the improvements in duration of performance in patients are not only associated with the rTMS but could, at least partially, be due to the effects of practice on a task administered 40 times over 20 days because similar improvements were also seen in absence of the rTMS in healthy volunteers.

### Accuracy of performance on the mCST

The pattern of results regarding the accuracy of performance on the mCST differed between patients and healthy volunteers according to comparison 1. Specifically, patients performed significantly more accurately (*p* = .038) on the last vs. the first mCST trial (Table 1 Accuracy). The same comparison was not statistically significant (*p* = .059) in the healthy volunteers.

Testing of comparison 2 showed that there were no significant main effects of TREATMENT (or BREAK) on the accuracy of performance on the mCST in both groups (Table 2 Accuracy; *p* = .632 in patients and *p* = .406 in healthy volunteers).

According to comparison 3, there was a significant main effect of BLOCK in both groups (Table 2 Accuracy). The patients performed the mCST significantly more accurately (*p* = .001) on BLOCK 2 (98.2 ± .3 s) vs. BLOCK 1 (96.2 ± .5 s) of the experiment regardless of treatment. Similarly, the healthy volunteers also performed significantly more accurately (*p* = .017) on BLOCK 2 (96.0 ± .7 s) vs. BLOCK 1 (94.2 ± 1.0 s) regardless of break.

When both TREATMENT (or BREAK) and BLOCK were taken into consideration, the pairwise comparisons showed that patients performed significantly more accurately on BLOCK 2 vs. BLOCK 1 before rTMS and on BLOCK 2 vs. BLOCK 1 after rTMS (Figure 1C; both comparisons with *p* = .004). The healthy volunteers performed significantly more accurately on BLOCK 2 vs. BLOCK 1 only after the break (Figure 1D; *p* = .032).

Finally, according to comparison 4, a difference in the accuracy of performance was observed in the two groups. Specifically, patients performed the mCST significantly more accurately (*p* < .001) on BLOCK 2 after rTMS vs. BLOCK 1 before rTMS (Table 2 Accuracy, main effect of TIME; Figure 1C). The same comparison was not statistically significant (*p* = .256) in the healthy volunteers.

Regardless of statistical significance, the same comparisons involving accuracy of performance on the mCST had similar effect sizes (partial eta squared, *η*^*2*^_*part*_) in both groups (Tables 1 and 2 Accuracy) except for comparison 4. Specifically, the patients performed the task more accurately on the last 10 days after rTMS vs. the first 10 days before rTMS with *p* < .001 and a high effect size (*η*^*2*^_*part*_ = .78). The same comparison was not statistically significant (*p* = .256) with a low effect size (*η*^*2*^_*part*_ = .18) in the healthy volunteers. Therefore, it appears that the improvement in accuracy on the last 10 days after rTMS vs. the first 10 days before rTMS was associated with the rTMS in patients because such an effect was not observed in healthy volunteers.

### Neurocognitive functioning and depression severity in patients

The neurocognitive functioning (RBANS scores) and depression scores (BDI, HAM-D) were compared before the first rTMS vs. after the last rTMS in patients using the non-parametric Wilcoxon Signed-Rank *T*-test due to violation of normality of scores in some of the variables included in these analyses. The results show that in addition to performing significantly faster and more accurately on the mCST (Table 1), the patients had significantly higher immediate memory scores (*p* = .030) and significantly lower depression scores on BDI (*p* = .046) and HAM-D (*p* = .019) after the last compared to before the first rTMS (Table 3).

**Table 3:** **Median RBANS and depression severity scores before the first rTMS (trial 1, day 1) vs. after the last rTMS (trial 8, day 20) in patients (*N*** **= 10)**

**Score (Median; range)**	**Day 1 before first rTMS**	**Day 20 after last rTMS**	***T (z)***	***p***_***two-tailed***_	*** r***
**RBANS**					
Immediate memory	96 (73–114)	104 (69–114)	2.5 (−2.2)	.030*	**-.49**
Delayed memory	94 (52–103)	96 (52–101)	10.0 (−1.1)	.262	-.24
Attention	97 (56–109)	106 (72–118)	13.5 (−1.4)	.150	-.31
Language	98 (78–117)	102 (87–120)	6.0 (−1.7)	.091	-.38
Visuospatial ability	86 (72–102)	82 (72–92)	10.5 (−1.7)	.083	-.38
**BDI**	28 (21–41)	18 (1–47)	8.0 (−2.0)	.046*	**-.45**
**HAM-D**	22 (19–29)	13 (1–31)	4.5 (−2.3)	.019*	**-.51**

*BDI*- Beck Depression Inventory; *HAM-D*- Hamilton Depression Rating Scale- 21 items; *RBANS*- Repeatable Battery for the Assessment of Neuropsychological Status; *T*- Wilcoxon Signed-Rank *T* Test. The effect size *r* was calculated as *r* = *z*/√*N*, where *N* = 20 (a total number of measurements/case).

*p < .05.

However, there were no correlations between the cognitive and clinical performance in the patients. Specifically, all Pearson Product Moment correlations (*N* = 10, two-tailed *p* < 0.05) between difference scores of variables listed in Table 3 (score after rTMS-before rTMS) vs. the mean accuracy or the mean duration of performance on the mCST (on all trials before and after rTMS) were non-significant with small coefficients *r* (results not shown).

## Discussion

The results of the current study suggest that the left frontal fast-frequency rTMS administered over 20 days is associated with improvements in some cognitive functions and reduction in depression severity in a pilot sample of treatment-resistant depression patients. Specifically, the concept-shifting ability, measured as accuracy of performance on the mCST, has improved on the last 10 days after the rTMS compared to the first 10 days before the rTMS in patients (Table 2). This improvement in patients was statistically significant and with a high effect size (.78) in contrast to the same comparison which was not significant and had a small effect size (.18) in healthy volunteers. Therefore, it can be speculated that the improvement in the task accuracy in patients was not due to practice alone following a multiple completion of the same task over 20 days because the same improvement was not observed in healthy volunteers. In contrast, all other cognitive improvements on the accuracy and duration of performance on the mCST were similar in both groups (patients and healthy volunteers) in terms of statistical significance and effect sizes and thus could not be specifically attributed to the rTMS.

In addition to improvement in the concept-shifting ability, an improvement in the immediate memory was seen after 20 days of rTMS compared to baseline in patients (Table 3). This result is in line with the improvement on a list-recall test following two weeks of a fast rTMS to the left DLPFC in patients with major depression [16]. The preliminary evidence from both studies suggests that the rTMS might be superior (or at least not worse) compared to the ECT in terms of not having the detrimental effects on memory that are seen after the ECT [16]. However, in general, it appears that the positive effects of rTMS on the cognitive functioning depend on the paradigms studied and the parameters of rTMS used and thus are not always observed [17].

In the current study it is unlikely that the performance on the mCST improved as a direct result of an improvement on the immediate memory since the scores on both tasks did not correlate. Instead of a cause-effect relationship, the improvement in both cognitive functions might be due to the direct or indirect (trans-synaptic) alteration of the DLPFC functioning. Specifically, the effect on memory, which is controlled by the left temporal cortex, suggests that the rTMS likely activates neural areas beyond the stimulation site either directly or indirectly by first activating the DLPFC which might have connections to these sites [18]. For instance, if the fast-frequency rTMS is mainly excitatory [17] it could reverse the reduced medial/middle prefrontal and hippocampal activity that are related to the positive affect in treatment-resistant depression [19]. On the other hand, if the fast-frequency rTMS has inhibitory properties [17] then it could reduce the temporal lobe hyperactivity that appears to be related to the negative affect disturbances in treatment-resistant depression [19]. The fast-frequency rTMS could also have transient effects, such as an increase in the neural activity during stimulation followed by hypoactivity [20]. In general, the changes in neural activity induced by the fast-frequency rTMS could result from increases in blood flow in regions implicated in depression, such as bilateral frontal, limbic and paralimbic regions [2], activation of the hypo-thalamo-pituitary axis indirectly measured by increases in serum thyroid-stimulating hormone, TSH [20], and/or stimulation of the prefrontal glutamatergic neurons at the stimulation site (left DLPFC) and also in remote brain regions, such as right DLPFC and left cingulated cortex [21].

The improvement in the cognitive functioning was accompanied by a reduction in severity of depression symptoms after the last rTMS compared to baseline in patients confirming the antidepressive properties of the left frontal high-frequency rTMS found in other studies [4]. It is difficult to speculate about the mechanism of reduction in depression symptoms following the rTMS based on the current data. One possibility could be that the rTMS alleviates depression and as a consequence improves cognitive functioning but the opposite could also hold true. On the other hand, the rTMS may independently alter both the cognitive ability and depression symptoms, perhaps by activating different neural pathways and regions. The last explanation appears possible since there were no significant correlations between cognitive functioning and depression scores in the current study.

One limitation of the current study is a lack of a matched clinical group and a matched healthy control group. Ideally, the current study should be expanded to test the performance on the mCST in patients receiving sham stimulation. However, the use of sham treatment in the rTMS is questionable because it appears that patients are able to correctly guess if they receive a real or a sham treatment based on their mood improvements experienced during the real rTMS [8]. It can be speculated that since not all cognitive functions improved following 20 days of the rTMS it is unlikely that the results in patients were due to placebo effect alone.

Another difficulty with the interpretation of results, seen not only in the current study but rather in most rTMS studies of treatment-resistant depression [2], is that the rTMS was used as an add-on treatment to a stable medication. Thus, the current improvement in cognitive functioning and reduction in severity of depression symptoms could be due to the combination of pharmacological treatment and the rTMS rather than the rTMS alone (assuming that placebo and/or practice effects were fully eliminated). In general, studies on patients receiving the rTMS monotherapy show the highest antidepressive properties (highest effect sizes) compared to studies using the rTMS as an add-on treatment [22]. Further studies on medication-free patients are needed to investigate the effects of the rTMS as a mono-therapy on various cognitive functions. Even though potentially confounding, a concurrent pharmacological treatment might be necessary to maintain the antidepressive effects of rTMS after cessation of rTMS [2].

Furthermore, the clinical significance of the improvements in the concept-shifting ability and the immediate memory were not investigated in the current study. In general, the prevalence of depression is high (approximately 11-15%) worldwide [23] and depression is associated with a high burden and social disability. It can only be speculated that the improvements in cognitive functioning associated with the rTMS contributed to at least some improvement in the well-being of the current patients. Since there was no follow-up, it is also unknown if the reduction in severity of symptoms and the improvements in cognitive functioning would persist and for how long following cessation of the rTMS treatment. In general, it appears that 1–4 weeks of the rTMS is well-tolerated but it is unclear if the therapeutic effects of treatment persist over time and if so, for how long [24].

## Conclusion

In conclusion, compared to baseline, improvements in immediate memory and reduction in severity of depression scores were observed following 20 days of left frontal fast-frequency rTMS in a pilot sample of patients with treatment-resistant depression. The novel result of the current study is that the rTMS is associated with an improvement in the concept-shifting ability using a task (mCST) that appears to be robust against the effects of practice. It can be speculated that these improvements are related to the direct or indirect activation of the DLPFC. The clinical significance of these results should be investigated in a larger sample of patients including a well-matched control group.

## Abbreviations

BDI: Beck depression inventory; DLPFC: Dorsolateral prefrontal cortex; ECT: Electroconvulsive therapy; HAM-D: Hamilton depression rating scale- 21 items; mCST: Modified concept shifting task; RBANS: Repeatable battery for the assessment of neuropsychological status; rTMS: Repetitive transcranial magnetic stimulation.

## Competing interests

The authors declare that they have no competing interest.

## Authors’ contributions

All authors read and approved the final manuscript; KK wrote the manuscript and performed all statistical analyses; all other authors collected the data and performed the preliminary analyses.

## Pre-publication history

The pre-publication history for this paper can be accessed here:

http://www.biomedcentral.com/1471-244X/12/163/prepub
